# Fecal Microbial Changes in Response to Finishing Pigs Directly Fed With Fermented Feed

**DOI:** 10.3389/fvets.2022.894909

**Published:** 2022-07-22

**Authors:** Xiaopeng Tang, Kai Zhang, Kangning Xiong

**Affiliations:** ^1^State Engineering Technology Institute for Karst Desertfication Control, School of Karst Science, Guizhou Normal University, Guiyang, China; ^2^College of Animal Science, Shanxi Agricultural University, Jinzhong, China

**Keywords:** fermented complete feed, fecal microbiome, genome prediction, microbial diversity, pigs

## Abstract

The present study investigated the effects of fermented complete feed (FCF) on fecal microbial composition during the grower-finisher period. A total of 20 pigs (Duroc × Landrace × Yorkshire, 48.74± 1.49 kg) were divided randomly into two groups: the CN group (pigs fed with a basal diet) and the FCF group (pigs fed with FCF). After a 60-day trial period, 3 pigs with middle-weight from each treatment were selected for fecal sampling and fecal microbiota analysis. The results showed that the FCF significantly increased operational taxonomic units (OUT) numbers, alpha diversity (Simpson index and Shannon index), and beta diversity, which means that FCF increased the fecal microbiota diversity. At the phylum level, the abundance of Tenericutes, Spirochaetae, Verrucomicrobia, and Cyanobacteria were changed in pigs fed with FCF; and at the genus level, the abundance of *Christensenellaceae_R-7_group, Treponema_2, Ruminococcaceae_UCG-005, Prevotellaceae_UCG-003, Phascolarctobacterium, Roseburia*, and *Prevotella_9* were changed in pigs fed with FCF. The linear discriminant analysis effect size (LEfSe) analysis showed that *Roseburia* and *Prevotella_9* genera were increased, while Tenericutes phyla and *Streptococcus, Christensenellaceae_R-7_group*, and *Lactobacillus* genera were decreased in the FCF group compared to the CN group. Phylogenetic Investigation of Communities by Reconstruction of Unobserved States (PICRUSt) results predicted that the relative abundance of infectious diseases: parasitic associated genes, xenobiotics biodegradation, and metabolism-associated genes were significantly reduced in the FCF group when compared with the CN group, and the relative abundance of signal transduction associated genes, amino acid metabolism-related genes, and replication and repair associated genes were significantly higher in the FCF group when compared with the CN group. In addition, the relative abundance of transport and catabolism-associated genes, membrane transport-associated genes, and biosynthesis of other secondary metabolite-associated genes tended to be higher in the FCF group when compared with the CN group; and the relative abundance of immune diseases associated genes tended to be lower in the FCF group when compared with the CN group. In conclusion, the FCF influenced the alpha and beta diversity of the fecal microbiota of pigs.

## Introduction

Gut microbiota is thought to be tightly associated with the intestinal barrier and plays a vital role in host health (1, 2). Maintaining the dynamic balance of intestinal microbiota is of prime importance to animal growth and health. However, many factors can cause microbial imbalances in the gastrointestinal tract, which may seriously affect nutrients' utilization and host health (3–5). Gut microbiota diversity and composition in humans as well in animals can be modulated by nutritional strategies, such as changes in dietary composition and the addition of feed additives (3, 6, 7). For example, Yin et al. (8) reported that a lysine-restricted diet affected the abundance of *Actinobacteria, Saccharibateria*, and *Synergistetes* in the gut of piglets. Adler et al. (9) showed that a dry feed significantly improved oral bacterial diversity when compared with a wet feed in cats. Wassie et al. (10) highlighted that dietary supplementation with *Enteromorpha* polysaccharide could modulate the cecal microbiota of broiler chickens. Therefore, nutritional regulation of the gut microbiota of animals may be an effective way to promote animal or host health.

Fermented feeds, which are easily decomposed biologically, contain abundant organic acids, amino acids, and small peptides, and have higher nutritional value than the original ingredients (11–13). For example, we showed in our previous study that, after fermentation with *Aspergillus Niger*, the contents of hydrolyzed protein and small molecular peptides in cottonseed meal were significantly increased, and the digestibility of amino acid and metabolic energy were also significantly increased in white leghorn roosters (14). The fermented feed has been suggested as one of the effective ways to improve the nutritional status as well as the intestinal morphology and microbiota composition of animals (11, 13). Previous studies reported that fermented feed can promote intestinal health by modulating the gut microbiota (15–17). Xie et al. (18) showed that a fermented soybean meal diet could improve the intestinal function by increasing the relative abundance of Firmicutes phylum and decreasing the relative abundance of Proteobacteria phylum in piglets. Similarly, Wang et al. (19) showed that fermented soybean meal could alleviate diarrhea symptoms in weaned piglets by modulating cecal microbiota composition (Proteobacteria phylum). Firmicutes includes a number of probiotics, such as *Lactobacillus* and *Clostridium*, which are beneficial to the intestinal barrier function (20, 21); while *Proteobacteria*, mainly composed of pathogenic microorganisms, such as *Escherichia coli* and *Campylobacter*, is strongly associated with intestinal inflammation (22, 23). So, the fermented feed has been shown to be beneficial in gut microbiota regulation. However, to our knowledge, the effects of fermented complete feed (FCF) on the fecal microbiota of finishing pigs are not known.

While the microbial composition is known to differ between the different intestinal segments, the fecal microbiome is most similar to the colon microbiome (23–25). Moreover, in pig production, fecal wastes are important pollution sources and the main carrier of zoonotic pathogens. Pathogenic bacteria in feces can contaminate the water, soil, and farm environments and can lead to human and animal infections (26, 27). The study of fecal microbiome can reflect the intestinal function as well as environmental pollution risks. Therefore, the aim of the present study was to study the effects of the FCF on fecal microbial diversity and composition during the grower-finisher period.

## Materials and Methods

### Animals, Diets, and Experimental Design

The basal diet was formulated according to the feeding standard of swine (NY/T 65-2004) to meet the nutritional requirement of the fatting pigs (Supplementary Table 1). The FCF preparation was the same as described in our previous study (28). Briefly, the complete feed was fermented at 24–34°C for 72 h in a ratio of 1:3:3:2 between *Lactobacillus plantarum*: *Candida utilis*: *Bacillus subtilis*: *Aspergillus niger*. After fermentation, the crude protein content (CP, 16.92%) and the crude fiber content (CF, 3.27%) were determined according to the AOCS (2009) method, and *Lactobacillus plantarum* (1.5 × 10^8^ Cfu/g), *Candida utilis* (2.1 × 10^6^ Cfu/g), and *Bacillus subtilis* (3.0 × 10^8^ Cfu/g) were measured using the plate counting techniques. The animal experimental procedure was the same as our previous study (28). Briefly, a total of 20 pigs (Duroc × Landrace × Yorkshire, 48.74 ± 1.49 kg) were randomly divided into the control group (pigs fed with a basal diet, CN) and the FCF group (pigs fed with an FCF diet), each treatment containing 10 pigs. All pigs were fed 3 times per day and had *ad libitum* access to water. The experiment lasted 60 days.

### Sample Collection

At the end of the feeding trial, three pigs with middle-weight from each treatment were selected for fecal samples collection. When pigs defecated, the fresh fecal was collected directly by hand, installed in cryopreserved tubes, then immediately frozen in liquid nitrogen, and stored at −80°C for microbiota analysis.

### Fecal Microflora Analysis

The fecal microbiota was analyzed by 16S high-throughput sequencing following the procedure described in our previous study (28). The main procedure included DNA extraction, PCR amplification of 16s rRNA, amplicon sequencing, and sequence data processing. Total genome DNA from fecal microbiota was extracted using the QIAamp DNA Stool Mini Kit (Qiagen GmbH, Hilden, Germany) following the manufacturer's instructions. The quality and quantity of extracted DNA were measured by reading the whole absorbing spectrum using a NanoDrop 2000 UV–vis spectrophotometer (Thermo Fisher Scientific, Waltham, MA, USA) and were used to calculate the DNA concentration and purity accordingly. DNA integrity was checked using 1% agarose gel electrophoresis. Illumina MiSeq sequencing and bioinformatics pipeline services were provided by a commercial company (Biomarker, Beijing, China). PCR amplified the V3–V4 region of the bacterial 16S rRNA gene to define the bacterial composition and abundance. The PCR amplicon was separated on 2% agarose gels, purified, pooled at equimolar concentrations, and finally subjected to paired-end sequencing on an Illumina MiSeq platform according to the standard methods. High-quality sequences were uploaded to QIIME (version 1.7.0) with effective tags clustered into operational taxonomic units (OTUs) using UCLUST in QIIME (version 1.7.0) at 97% sequence identity. The OTUs were annotated based on Silva (bacteria) taxonomy databases. Then, the microbiome composition of each sample was calculated at each level (phylum, class, order, family, genus, species), and QIIME software was used to generate species abundance tables at different classification levels.

Alpha and beta diversity analyses further determined the composition and abundance of the microflora. The linear discriminant analysis effect size (LEfSe) analysis was conducted by LEfSe software to identify the different abundant taxa between the CN group and FCF group. OTUs were further used for genome prediction of microbial communities by Phylogenetic Investigation of Communities by Reconstruction of Unobserved States (PICRUSt).

### Statistical Analysis

Data were presented as the mean ± standard error of the mean (SEM). The data were subjected to the unpaired *t*-test using SPSS 21.0 programs (SPSS, Inc., Chicago, IL, USA). A *p* < 0.05 was considered to be statistically significant.

## Results

### Growth Performance

The effects of FCF on the growth performance of pigs were reported in our previous study (28). Briefly, pigs fed with FCF exhibit a higher (*P* < 0.05) final body weight (FBW), average daily gain (ADG), and average daily feed intake (ADFI), and a lower (*P* < 0.05) feed-to-gain ratio (F/G) compared to pigs fed with a basal diet (Supplementary Table 2).

### Data Quality Evaluation of Fecal Microflora Sequencing

The data quality was primarily assessed through PE reads, raw tags, clean tags, effective tags, sequence length, and effective (%). The result revealed that an average of 79,998.67 and 79976.00 PE reads were generated from the CN and FCF groups, respectively (Table 1). FCF did not affect the raw tags, clean tags, effective tags, effective (%), and average length.

**Table 1:** Data quality evaluation of fecal microflora sequencing.

**Item**	**CN**	**FCF**	**SEM**	***P*-Value**
PE reads	79,998.67	79,976.00	104.02	0.927
Raw tags	77,766.33	77,809.00	142.78	0.900
Clean tags	68,297.00	68,601.33	242.62	0.590
Effective tags	65,744.33	66,631.00	517.93	0.454
Average length	415.67	417.00	0.42	0.116
Effective (%)	82.21	83.28	0.55	0.391

*Values are expressed as mean ± SEM, n = 3; CN: Control group (pigs received a basal diet); FCF, fermented complete feed group (pigs received fermented complete feed). A p < 0.05 indicated statistical significance*.

### OTUs Analysis

**Figure 1:**
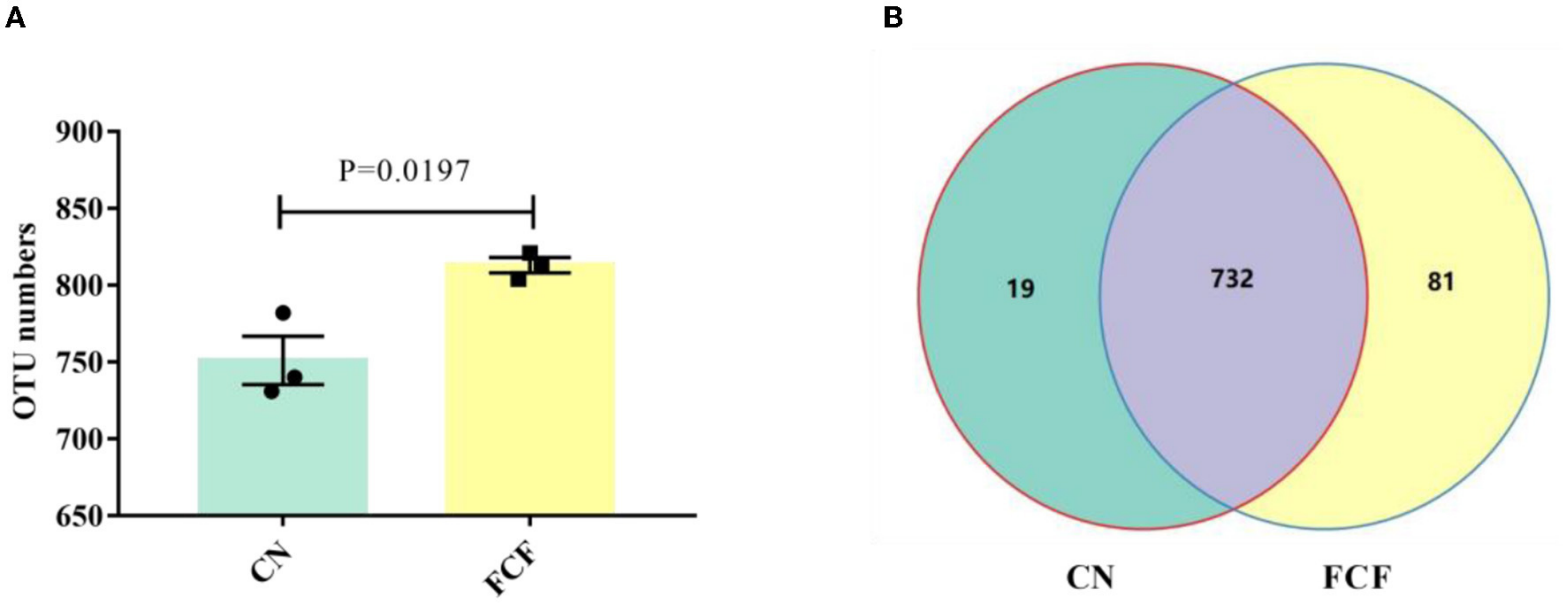
Effects of fermented complete feed on an operational taxonomic unit (OTUs) in fecal samples. Values are expressed as mean ± SEM, *n* = 3. **(A)** The number of observed OTUs between CN and FCF groups; **(B)** Venn diagram of OTUs; Control group; FCF, fermented complete feed group. A *p* < 0.05 was taken to indicate statistical significance.

The effects of FCF on OTUs in fecal samples are illustrated in Figure 1. The results showed that an average of 751 OTUs were identified in the CN group, and an average of 813 OTUs were identified in the FCF group (*P* < 0.05; Figure 1A). Based on the OTU classification, we calculated the same number of OTUs between the CN group and the FCF group. The Venn diagrams showed that 732 OTUs were shared between the CN group and the FCF group (Figure 1B). Only 19 and 81 OTUs were unique in the CN and FCF groups, respectively.

### Alpha Diversity

**Figure 2:**
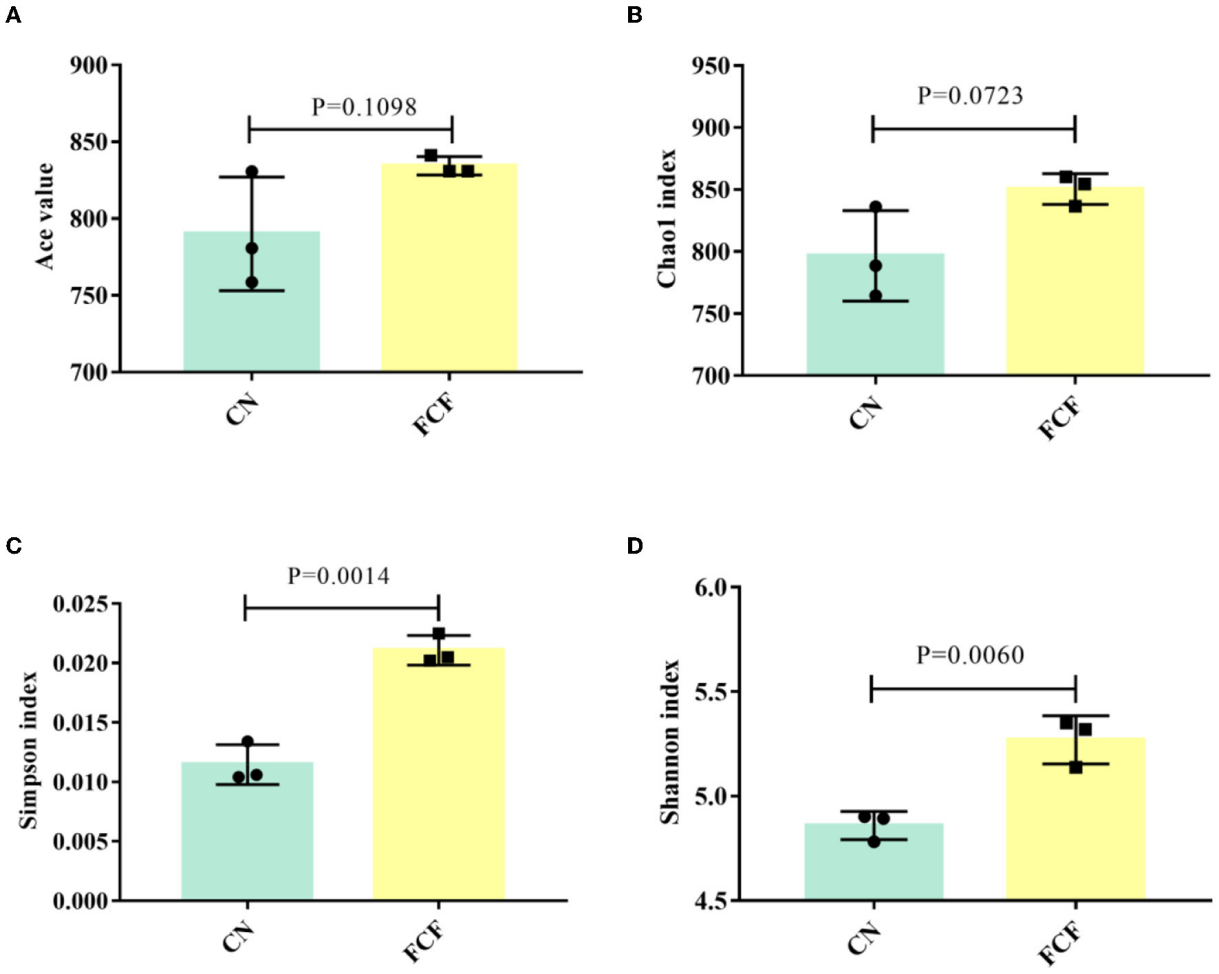
The alpha diversity of the fecal microbial community in pigs fed with a basal diet (CN) or fermented complete feed (FCF). Values are expressed as mean ± SEM, *n* = 3. **(A)** ACE index; **(B)** Chao1 index; **(C)** Simpson index; **(D)** Shannon index. A *p* < 0.05 was taken to indicate statistical significance.

Alpha diversity including the ACE index, the Chao1 index, the Shannon index, and the Simpson index are shown in Figure 2. The results showed that FCF significantly increased (*P* < 0.05) the Simpson index (Figure 2C) and the Shannon index (Figure 2D), and FCF tended to increase (*P* = 0.0723) the Chao1 index (Figure 2B).

### Microbiota Composition at the Phylum Level

**Figure 3:**
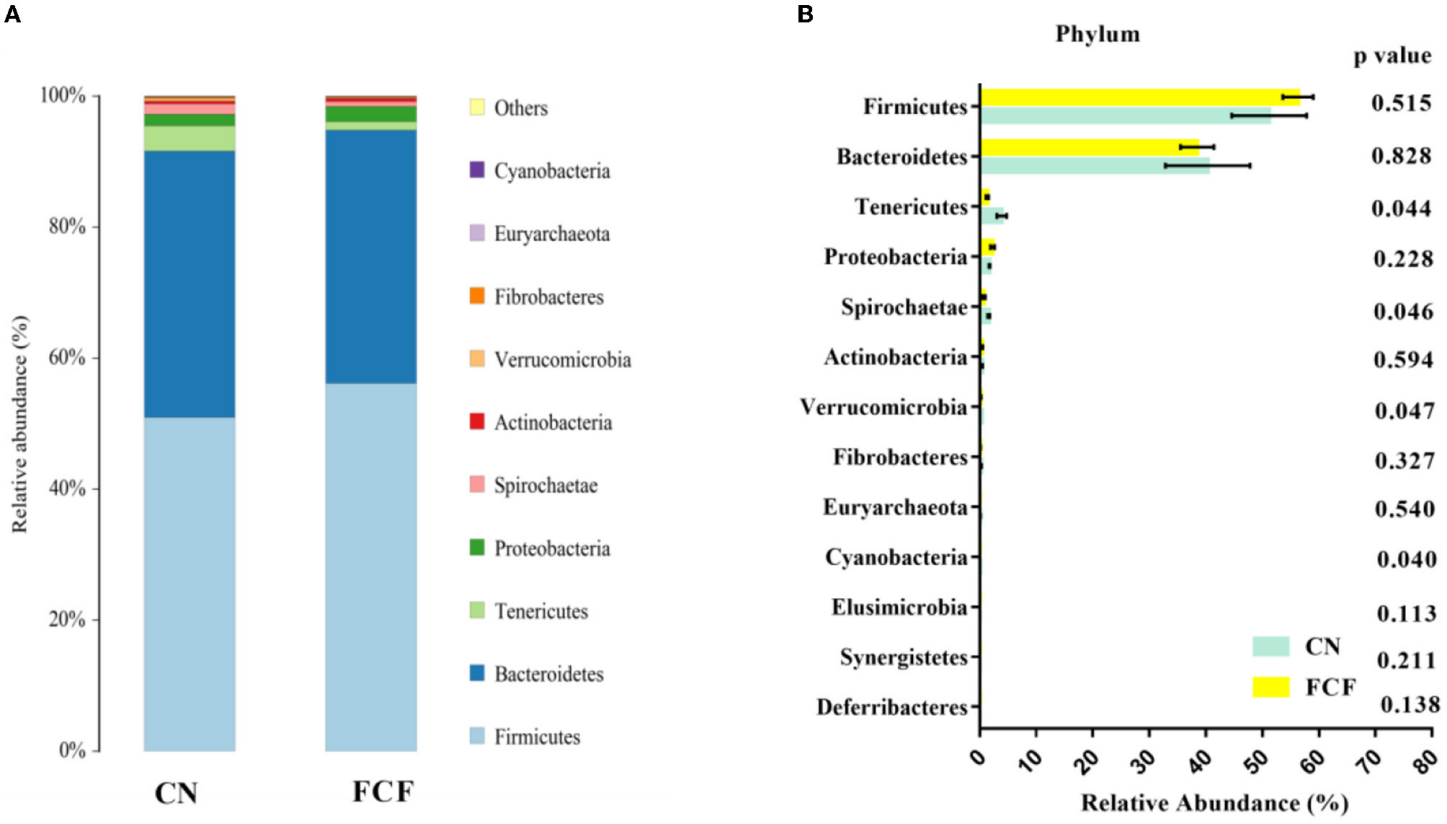
The relative abundance of major phylum in response to pigs fed with a basal diet (CN) or fermented complete feed (FCF). Values are expressed as mean ± SEM, *n* = 3. **(A)** Relative abundance of bacterial communities at the phylum level; **(B)** Unpaired *t*-test analysis of the difference between CN and FCF group. A *p* < 0.05 was taken to indicate statistical significance.

The effects of FCF on fecal microbiota composition at the phylum level are presented in Figure 3. Top 10 phyla were identified in the fecal samples, of which Firmicutes, Bacteroidetes, Tenericutes, Proteobacteria, and Spirochaetae comprised the dominant community members (Figure 3A). The abovementioned phyla accounted for 98.86% of the microbial population in the CN group and 99.17% in the FCF group (Figure 3B). Figure 3B shows that pigs fed with FCF have a lower (*P* < 0.05) abundance of Tenericutes, Spirochaetae, Verrucomicrobia, and Cyanobacteria in fecal samples when compared with pigs fed with a basal diet.

### Microbiota Composition at the Genus Level

The effects of FCF on the genus-level composition of fecal microbiota are presented in Table 2. *Bacterium* and *Prevotellaceae_NK3B31_group* were observed to be the two most abundant genera, accounting for 38.44% and 30% of the microbial population in the CN and FCF groups, respectively. Furthermore, the abundance of *Christensenellaceae_R-7_group* and *Treponema_2* were significantly reduced (*P* < 0.05) in pigs fed with FCF than in those fed with a basal diet, whereas *Ruminococcaceae_UCG-005, Prevotellaceae_UCG-003, Phascolarctobacterium, Roseburia*, and *Prevotella_9* were found abundantly (*P* < 0.05) in pigs fed with FCF than in those fed with a basal diet. Upon comparing to the pigs fed with a basal diet, the pigs fed with FCF tended to decrease (*P* = 0.059) the abundance of *Bacterium* and tended to increase the abundance of *Streptococcus* (*P* = 0.081) and *Alloprevotella* (*P* = 0.097).

**Table 2:** The relative abundance of the major genus in response to pigs fed with fermented complete feed.

**Item**	**CN**	**FCF**	**SEM**	***P*-Value**
Bacterium	28.9630	23.0123	1.6752	0.059
Prevotellaceae_NK3B31_group	9.4816	7.8805	1.1112	0.534
Ruminococcaceae_UCG-002	5.2590	3.9341	0.4874	0.201
Christensenellaceae_R-7_group	4.3087	1.4302	0.0604	0.002
Lactobacillus	4.2478	0.7280	1.5720	0.312
Lachnospiraceae_XPB1014_group	3.8265	2.2147	0.0689	0.334
Rikenellaceae_RC9_gut_group	3.5922	3.6759	0.4036	0.930
Streptococcus	3.4790	0.9127	0.7568	0.081
Ruminococcaceae_UCG-005	2.8437	4.4626	0.4345	0.039
Ruminococcaceae_NK4A214_group	2.5467	2.7782	0.0540	0.730
[Eubacterium]_coprostanoligenes_group	2.2936	2.2313	0.1041	0.801
Prevotella_1	1.9970	3.004	0.4629	0.328
Treponema_2	1.6190	0.7311	0.2425	0.046
Peptoclostridium	1.3694	1.9278	0.1865	0.146
Clostridium_sensu_stricto_1	1.3603	1.5407	0.1648	0.640
Lachnospiraceae_AC2044_group	1.2392	1.7101	0.1864	0.243
Ruminococcaceae_UCG-010	1.1858	0.9610	0.0850	0.216
Prevotellaceae_UCG-003	1.1493	2.0682	0.2143	0.002
Phascolarctobacterium	1.0606	2.6766	0.4452	0.049
Oscillospira	1.0272	1.2670	0.2222	0.645
Parabacteroides	1.0168	0.7277	0.1159	0.250
Alloprevotella	0.5847	1.3786	0.2422	0.097
Roseburia	0.4662	3.8016	0.7809	0.003
Prevotella_9	0.1977	2.2238	0.5031	0.014

*The genus (in the CN group or the FCF group) >1% are presented. Values are expressed as mean ± SEM, n = 3; CN: Control group; FCF, fermented complete feed group. A p < 0.05 indicated statistical significance*.

### Beta Diversity

**Figure 4:**
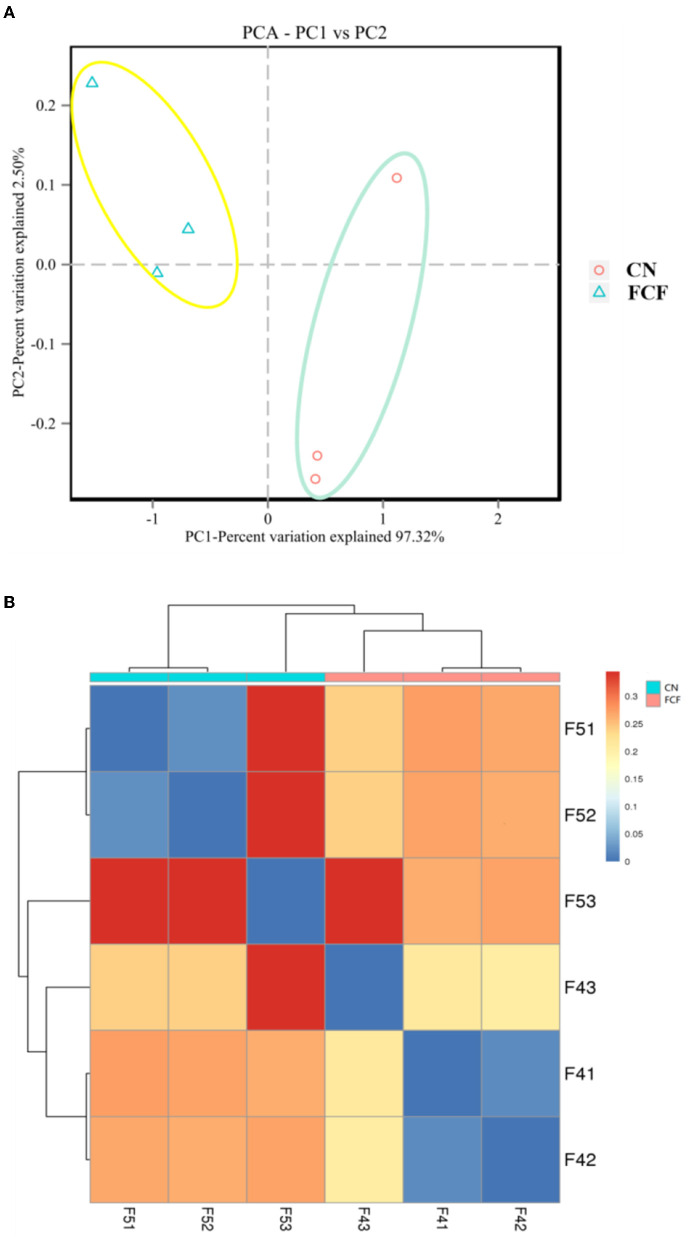
The beta diversity of the fecal microbial community in pigs fed with a basal diet (CN) or fermented complete feed (FCF) (*n* = 3). **(A)** Cluster analysis by principal component analysis (PCA); **(B)** Cluster analysis by a heatmap, the color gradient from blue to red indicates the distance from near to far between samples.

Principal component analysis (PCA) and beta-diversity of distance matrix visualized on a heatmap used Weighted UniFrac distances to estimate the bacterial community's overall differences. The growing microbial community in the FCF group exhibited a distinct clustering. In contrast, the CN group showed a scattered distribution structure in the PCA analysis (Figure 4A). The heatmap chart's color gradient from blue to red represents the distance between samples. The chart showed that the samples in the CN group (F51, F52, F53) are farther apart than that in the FCF group (Figure 4B).

### Linear Discriminant Analysis (LDA) Effect Size Analysis

**Figure 5:**
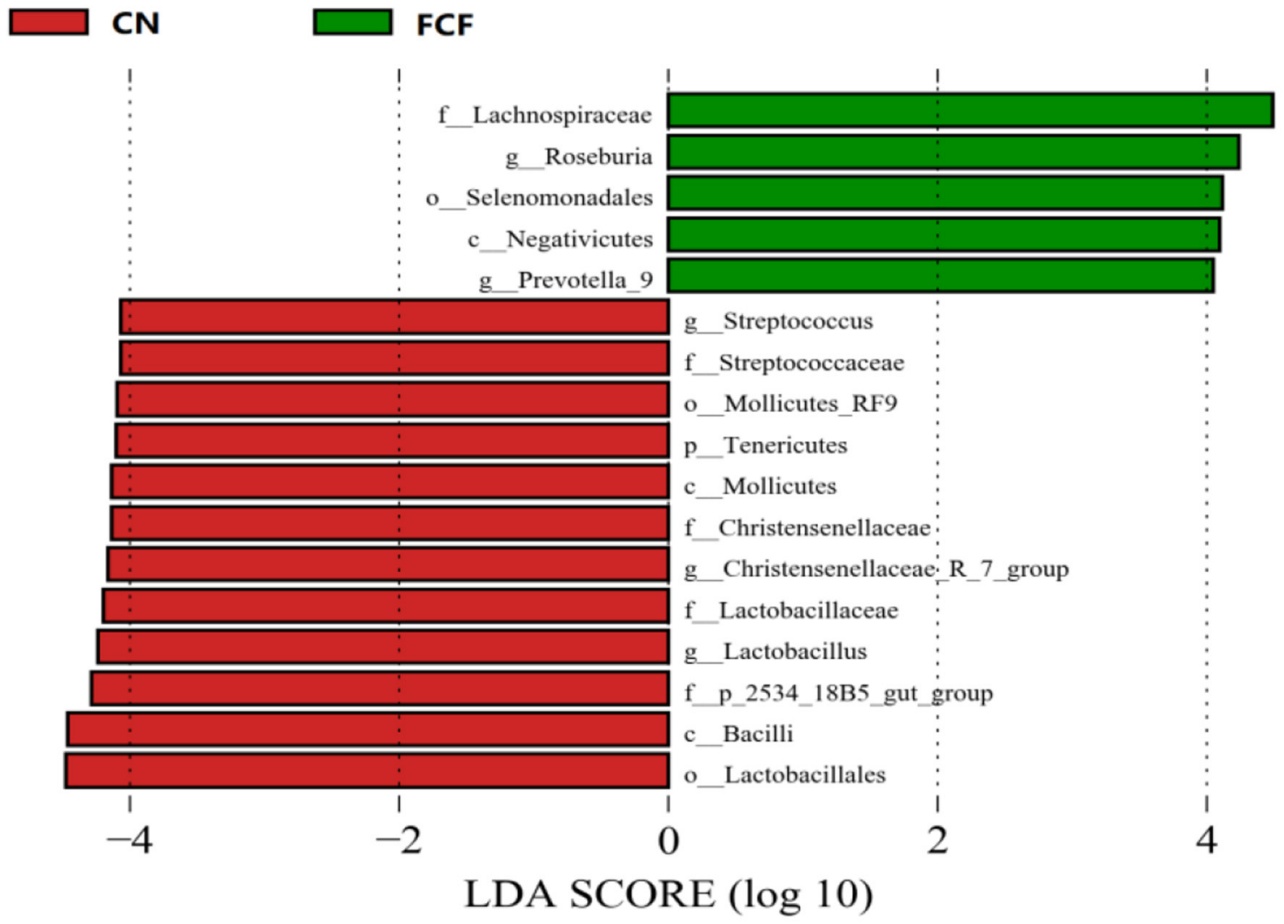
LEfSe analysis filtered out the biomarkers of the microbial community after pigs received basal diet (CN) or fermented complete feed (FCF) (*n* = 3). Red bars (negative LDA scores) represent bacteria that are more abundant in CN fecal samples than in FCF. Green bars (positive LDA scores) represent bacteria that are more abundant in FCF fecal samples than in CN fecal samples.

LEfSe package was used to identify the microbial taxa that are differentially abundant across the CN and FCF groups. LEfSe analysis revealed that Tenericutes phylum and *Streptococcus, Christensenellaceae_R-7_group*, and *Lactobacillus* genera exhibit lower abundance, which was decreased in the FCF group when compared with the CN group. In contrast, *Roseburia and Prevotella_9* genera were abundant in the FCF group (Figure 5).

### Functional Genes Prediction

**Figure 6:**
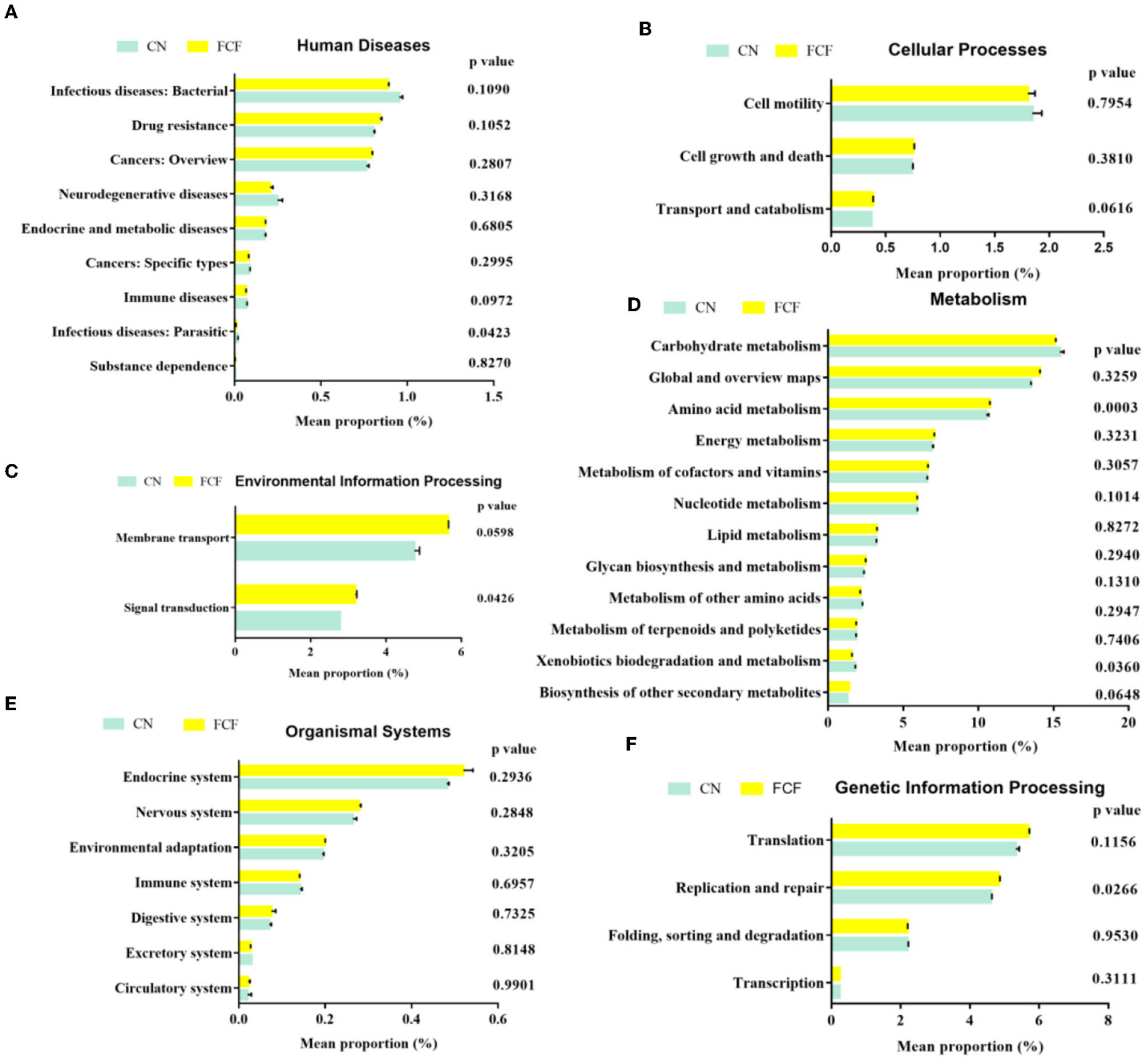
Predictive functional profiling of microbial communities by PICRUSt. Values are expressed as mean ± SEM, *n* = 3. **(A)** Human diseases; **(B)** Cellular processes; **(C)** Environmental information processing; **(D)** Metabolism; **(E)** Organismal systems; **(F)** Genetic information processing. CN, Control group; FCF, fermented complete feed group. A *p* < 0.05 was taken to indicate statistical significance.

OTUs were used for generating functional predictions of microbial communities by PICRUSt. The findings revealed the relative abundance of infectious diseases: parasitic genes in bacteria associated with human diseases were significantly reduced in the FCF group when compared with the CN group (*P* = 0.0423). The relative abundance of immune disorder-related genes is tended to decrease (*P* = 0.0972) greatly in pigs fed with an FCF diet (Figure 6A). In cellular processes, pigs fed with an FCF diet tended (*P* = 0.0616) to increase the transport and catabolism-associated gene expression (Figure 6B). Moreover, the relative abundance of signal transduction-associated genes (*P* = 0.0426) and membrane transport-associated genes (*P* = 0.0598) were significantly higher in the FCF group than in those of the CN group (Figure 6C). Pertaining to metabolism, the pigs fed with an FCF diet had abundant amino acid metabolism-associated genes (*P* = 0.0003). On the contrary, a decreasing trend was observed in the relative abundance of xenobiotics biodegradation and metabolism-related genes (*P* = 0.0360). In addition, a greater tendency (*P* = 0.0972) was observed for the biosynthesis of other secondary metabolite-associated genes (Figure 6D) compared to the pigs fed with a basal diet. There was no effect on bacterial genes related to organismal systems when pigs were fed with an FCF diet (Figure 6E). For genetic information processing, replication and repair associated genes were significantly increased (*P* = 0.0266) in pigs fed with FCF (Figure 6F).

## Discussion

The gut microbiota maintains a symbiotic relationship with the host and regulates several important functions including host metabolism, nutrients digestion and absorption, immunity, and intestinal barrier function (5, 29). So, the dynamic balance of intestinal microbiota is crucial for animal intestinal health, while optimum intestinal health is crucial to animal growth and health (30, 31). Growing shreds of evidence demonstrated that many factors including diets can affect the gut microbiota community and function (6, 7, 29, 32, 33). For instance, Guo et al. (34) illustrated that a high-fat diet exacerbates the pro-inflammatory cytokine-associated gut microbiota in mice. Spring et al. (35) reported that a low–protein diet significantly increased the abundance of *Christensenedilaceae, Aligiphilus*, and *Algoriphagus* genera and decreased the relative abundance of *Prevotella* genus in pigs. The fecal microbiome exhibit a high degree of resemblance with the colon microbiome (23, 24), due to its widespread availability, and fecal microbes are frequently tested to reflect the status of gut microbes. In the present study, we studied the effects of FCF on the fecal microbiota of pigs during the grower-finisher period and observed a change in fecal microbiota in response to FCF diet intake. It is commonly accepted that pigs share similar anatomic, physiologic, and metabolic characteristics with humans (36); however, the findings in the present study can also provide new insight into the influences of fermented food on humans' gut microbiome.

Previous studies showed that fermented feed can alter the gut microbiota structure and composition during different growth stages in pigs (9, 16, 28). In this study, 16S high-throughput sequencing technique was applied to detect the fecal bacteria diversity and compositions of pigs fed with an FCF diet. The results showed that the FCF group had a higher OTU number than the CN group, indicating that FCF elevated the fecal microbial diversity. The richness and diversity of the microbial community can reflect the redundancy of the gut bacterial species (37). Alpha diversity (ACE index, Chao1 index, Shannon index and Simpson index) has been hailed as a comprehensive indicator of species richness and evenness (21, 38). In the present study, the comparative analysis revealed a higher Shannon index and Simpson index for the FCF group than for the CN group, which is concordant with our previous study (28). Pigs fed FCF exhibited higher Shannon index and Simpson index in the jejunum and the cecum and higher ACE index, Chao1 index, Shannon index, and Simpson index in the cecum (28). It was confirmed with an interpretation from our findings that FCF increased the community stability and strengthened the connections between communities of fecal microbes. Beta diversity can reflect the species diversity between communities (21). In the current study, the PCA cluster analysis and heatmap used Weighted UniFrac distances analysis revealed that the samples in the FCF group represent a distinct clustering of microbial community structure. In contrast, the samples in the CN group showed a scattered distribution structure. The heatmap chart analysis revealed that the samples in the CN group are farther apart than those in the FCF group, signifying the FCF-driven alteration of the beta diversity of microorganisms.

The fecal microbial composition was analyzed at the phylum and genus levels. In general, Firmicutes and Bacteroidetes are the most predominant phyla in a healthy gut (39, 40). Our findings are consistent with previous studies and confirmed that Firmicutes and Bacteroidetes phyla are the most dominant bacteria community members both in CN and FCF groups. Firmicutes and Bacteroidetes were considered potentially beneficial autochthonous bacteria in animal intestinal tracts (41), which play an important role in improving intestinal homeostasis, such as reducing intestinal diseases and promoting the production of short-chain fatty acids (42, 43). At the genus level, the dominant bacteria differs among different studies. Quan et al. (44) reported that *Streptococcus, Clostridium sensu stricto 1*, and *Lactobacillus* were the three most predominant genera in the feces of pigs. According to Wang et al. (45), *Prevotella* and *Clostridium sensu stricto 1* were the two most abundant genera in the feces of pigs. In the present study, *Bacterium* and *Prevotellaceae_NK3B31_group* were the two most abundant genera accounting for 38.44 and 30.89% of the microbial population in the CN and FCF groups, respectively. Altering the diet plan is likely to change the composition of gut microbiota (29, 32, 33). The current study revealed the fact that administration of FCF diets significantly changes the abundance of microbiota, in which the most prominent are Tenericutes, Spirochaetae, Verrucomicrobia, and Cyanobacteria phyla and *Christensenellaceae R-7 group, Treponema 2, Ruminococcaceae UCG-005, Prevotellaceae UCG-003, Phascolarctobacterium, Roseburia*, and *Prevotella_9* genera in the feces of pigs. One of the possible reasons of FCF affecting the composition of the fecal microbiota is the existence of *Aspergillus niger*, a fungus with strong enzyme (cellulase, protease, and amylase) secreting ability (14), which renders the decomposition of macromolecules into small molecules, thus improving the palatability and nutrient digestibility of feed (28). The increased nutritional intake provides sufficient nutrients for gut microbiota, thereby altering the fecal microbial abundance and composition. Another possible reason is that the FCF diets contain *Lactobacillus plantarum, Candida utilis*, and *Bacillus subtilis* (28), which can function as probiotics (46–49), to affect the fecal microbial abundance and composition.

PICRUSt was employed to predict the functional composition of a metagenome using marker genes from 16S rRNA sequencing (50). PICRUSt analysis predicted that FCF could reduce the risk of parasitic infection by reducing the relative abundance of infectious diseases: parasitic-associated genes; FCF could improve signal transduction by increasing the relative abundance of associated genes. Similarly, FCF enhanced amino acid metabolism and reduced xenobiotics biodegradation and metabolism, indicating the beneficial effect of CFC feeding on pig metabolism. Moreover, FCF potentially improved the ability of replication and repair. Previous studies demonstrated that fermented feed can improve not only growth performance but also animal health (12, 28, 51–54). In agreement, the current study demonstrated that FCF could improve the growth potential and health status of pigs. It is possible that FCF alteration of intestinal microbiota affected intestinal bacterial gene expression, such as infectious diseases: parasitic, transport and catabolism, signal transduction, amino acid metabolism, xenobiotics biodegradation, and metabolism, and replication and repair, and ultimately promoted animal health.

## Conclusions

The current study showed that FCF significantly increased the fecal bacteria diversity, which is indicated by the increased OUT numbers, alpha diversity (Simpson index and Shannon index), and beta diversity of pigs. At the phylum level, FCF significantly affected the fecal abundance of Tenericutes, Spirochaetae, Verrucomicrobia, and Cyanobacteria; At the genus level, *Christensenellaceae_R-7_group, Treponema_2, Ruminococcaceae_UCG-005, Prevotellaceae_UCG-003, Phascolarctobacterium, Roseburia*, and *Prevotella_9* were changed in response to FCF. Predictive functional profiling of microbial communities by PICRUSt predicted that an altered intestinal microbiome caused by FCF might influence infectious diseases: parasitic, transport and catabolism, signal transduction, amino acid metabolism, xenobiotics biodegradation and metabolism, and replication and repair. In conclusion, FCF modulated the fecal microbial abundance and composition of pigs.

## Data Availability Statement

The datasets presented in this study can be found in online repositories. The names of the repository/repositories and accession number(s) can be found at: https://www.ncbi.nlm.nih.gov/, PRJNA787021.

## Ethics Statement

The experimental procedures involving animals were reviewed and approved by the animal welfare committee of the Shanxi Agricultural University (Taigu, China) with an ethic approval number SXAU-2018-0093.

## Author Contributions

XT and KZ performed the experiments, contributed to experimental concepts and design, provided scientific direction, and finalized the manuscript. XT, KX, and KZ performed the statistical analyses and wrote the manuscript. All authors read and approved the final manuscript.

## Funding

This research was funded by grants from the Key Project of Science and Technology Program of Guizhou Province (Grant No. 5411 2017 Qiankehe Pingtai Rencai); the World Top Discipline Program of Guizhou Province (Grant No. 125 2019 Qianjiao Keyan Fa); Guizhou Normal University Academic New Seedling Fund project [Qianshi Xinmiao (2021)B16]; the Key Research and Development Program of Shanxi Province (201603D221026-4); the Central Guide Local Science and Technology Development Special Fund (2017GA630002); and the Natural Science Research Project of Education Department of Guizhou Province [Qianjiaohe KY Zi (2021) 294].

## Conflict of Interest

The authors declare that the research was conducted in the absence of any commercial or financial relationships that could be construed as a potential conflict of interest.

## Publisher's Note

All claims expressed in this article are solely those of the authors and do not necessarily represent those of their affiliated organizations, or those of the publisher, the editors and the reviewers. Any product that may be evaluated in this article, or claim that may be made by its manufacturer, is not guaranteed or endorsed by the publisher.
