# Supplementary material for: Fecal Microbial Changes in Response to Finishing Pigs Directly Fed With Fermented Feed
Source: Front Vet Sci. 2022 Jul 22;9:894909. doi: 10.3389/fvets.2022.894909 (PMC9354886; doi:10.3389/fvets.2022.894909)
Supplement: Supplementary file 1 [file Table_1.docx]

**Table S1** Composition and nutrient level of basal diet

| Ingredient | Content (%) | Nutrient levels^b^ | Content (%) |
| --- | --- | --- | --- |
| Corn | 76.3 | Digestible energy, MJ/kg | 14.26 |
| Soybean meal | 16.5 | Crude protein | 15.89 |
| Soybean oil | 0.56 | Crude fiber | 3.45 |
| Corn starch | 2.44 | Crude Fat | 5.94 |
| CaHPO_4_ | 0.65 | Calcium | 0.62 |
| Calcium carbonate | 1.08 | Total phosphorus | 0.42 |
| Salt | 0.43 | Available phosphorus | 0.20 |
| Lysine | 0.16 |  |  |
| Premix ^a^ | 1.75 |  |  |
| Total | 100 |  |  |

^a^ Premix for 1 kg of complete diet contained: Cu as copper sulfate,10 mg; Fe as iron sulfate, 100 mg; Se as sodium selenite, 0.30 mg; Zn as zinc oxide, 100 mg; Mn as manganese oxide, 10 mg; vitamin D 3, 386 IU; Vitamin A as retinyl acetate, 3086 IU; Vitamin E as D-tocopherol, 15.4 IU; Vitamin K as menadione sodium bisulfate, 2.3 mg; Vitamin B2, 3.9 mg; Calcium pantothenate, 15.4 mg; Niacin, 23 mg; Vitamin B12, 15.4 mg.

^b^ DE was a calculated value. The other nutrient levels were measured values.
