# Supplementary material for: Fecal Microbial Changes in Response to Finishing Pigs Directly Fed With Fermented Feed
Source: Front Vet Sci. 2022 Jul 22;9:894909. doi: 10.3389/fvets.2022.894909 (PMC9354886; doi:10.3389/fvets.2022.894909)
Supplement: Supplementary file 2 [file Table_2.DOCX]

**TABLE S2** Effects of fermented complete feed on growth performance of pigs

| Item | CN | FCF | *P*-value |
| --- | --- | --- | --- |
| Initial body weight (kg) | 48.52±1.53 | 48.97±1.34 | 0.327 |
| Final body weight (kg) | 100.34±4.48 | 104.56±7.76 | 0.031 |
| ADG (kg/d) | 0.86±0.05 | 0.93±0.01 | 0.045 |
| ADFI (kg/d) | 2.72±0.11 | 2.85±0.21 | 0.028 |
| F/G | 3.16±0.15 | 3.06±0.19 | 0.037 |

Values are expressed as means ± SE; CN: Control group, pigs received a basal diet; FCF: fermented complete feed group, pigs received fermented complete feed.
